# The effects of interleukin-21 in the biology of transplant rejection

**DOI:** 10.3389/fimmu.2025.1571828

**Published:** 2025-05-01

**Authors:** Xiandong Zeng, Yixiao Pan, Qiang Xia, Kang He

**Affiliations:** ^1^ Department of Liver Surgery and Liver Transplantation, Renji Hospital, School of Medicine, Shanghai Jiao Tong University, Shanghai, China; ^2^ Shanghai Engineering Research Center of Transplantation and Immunology, Shanghai, China

**Keywords:** interleukin-21 (IL-21), T lymphocyte, B lymphocyte, transplant rejection, antibody-mediated rejection (AMR)

## Abstract

Interleukin-21 (IL-21) is a cytokine that plays a crucial role in regulating immune responses, affecting various immune cell types, including T cells, B cells, natural killer (NK) cells, and dendritic cells. IL-21 is primarily produced by CD4+ T cells, particularly follicular helper T (Tfh) cells and Th17 cells, and has been shown to be extensively involved in regulating both innate and adaptive immunity. IL-21 is particularly significant in the differentiation, proliferation, and effector functions of T cells and B cells. In the context of organ transplantation, IL-21 contributes to the promotion of acute transplant rejection and the development of chronic rejection, which is primarily antibody-mediated. This review summarizes relevant studies on IL-21 and discusses its multifaceted roles in transplant immune rejection, providing insights into therapeutic strategies for either inhibiting graft rejection or promoting tolerance. It also explores the feasibility of blocking the IL-21 signaling pathway within current immunosuppressive regimens, aiming to provide further clinical references.

## 1 Introduction

Organ transplantation is the only viable therapeutic option for patients with end-stage conditions such as cirrhosis and renal failure. In allogeneic organ transplantation, polymorphisms in the MHC genes between individuals facilitate immune cell recognition of “foreign” components, initiating immune signaling between the donor and recipient. This process triggers an immune response against the donor graft, which carries “foreign” antigens, ultimately leading to transplant rejection. In innate immunity, NK cell infiltration into the graft followed by the release of granzyme B and interferon-γ is considered one of the hallmarks of acute rejection (1, 2). Macrophages migrate to the local environment of the graft and exert phagocytic activity (3), and they can also function as antigen-presenting cells (APCs) to activate T cells (4), releasing various inflammatory cytokines (e.g., IL-1, IL-6, TNF-α) to amplify the immune response (5). In adaptive immunity, T cell-mediated rejection (TCMR), which involves cytotoxic CD8+ T cell responses against mismatched MHC class I allogeneic antigens, is considered the core mechanism of acute transplant rejection (6, 7). This process induces target cell apoptosis via the release of perforin and granzyme B or through the Fas/FasL pathway. Antibody-mediated rejection (AMR) is considered the primary pathway for chronic graft injury and dysfunction, where donor-specific antibodies (DSA) secreted by plasma cells lead to chronic vascular changes in the graft. For instance, in liver transplantation, DSA can cause occlusive arterial lesions (8, 9), and in lung transplantation, it leads to intimal thickening of pulmonary arteriovenous vessels (10–12). In summary, transplant rejection is a dynamic and complex process mediated by both innate and adaptive immunity. Various immune cells utilize different mechanisms to recognize and attack the donor graft, leading to both acute rejection and chronic graft injury and dysfunction.

**Figure 1:**
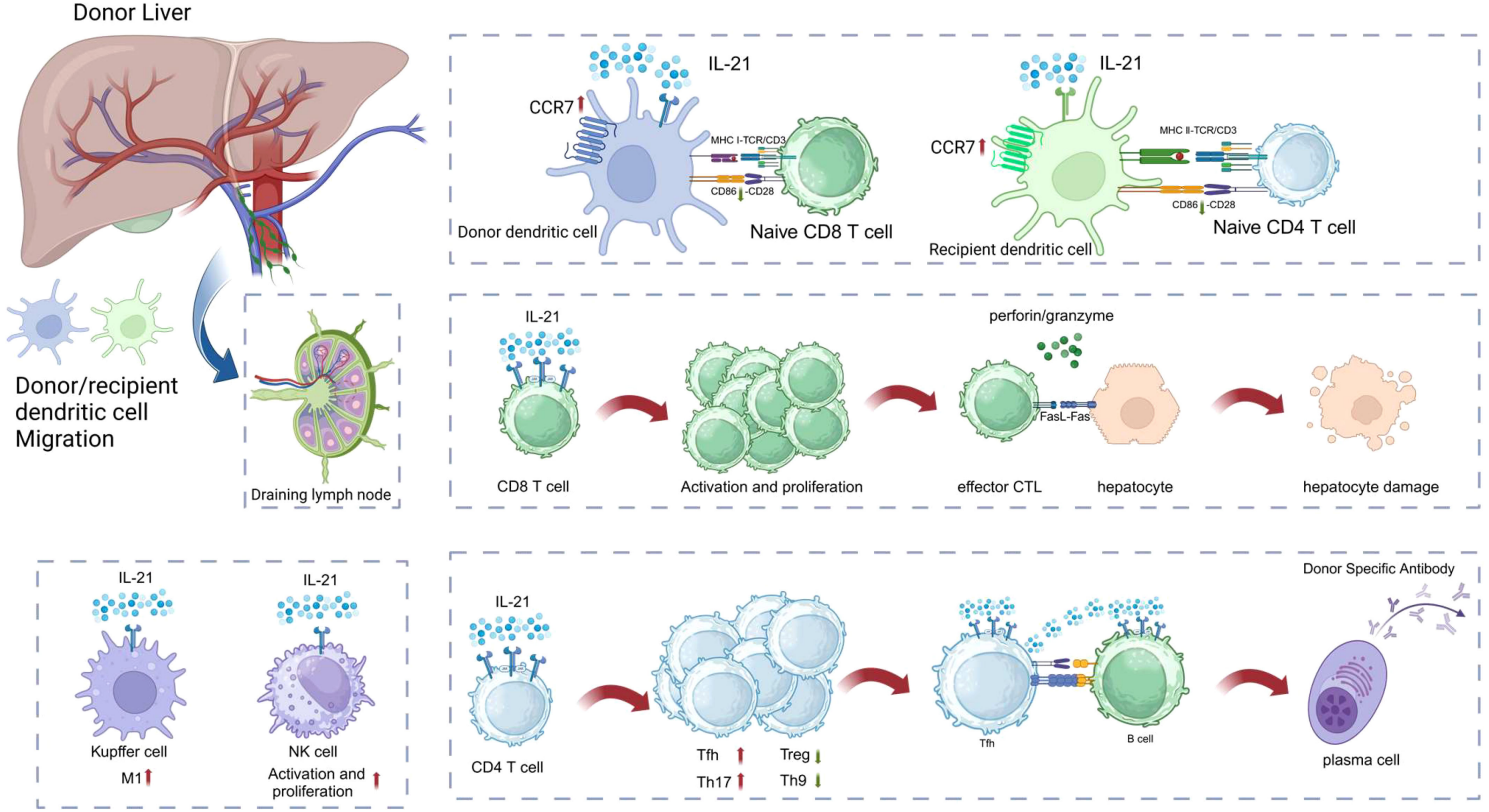
IL-21 affects a variety of immune cell effector functions, deeply involved in organ transplantation rejection. The Figure was created with www.biorender.com.

Cytokines profoundly influence the fate of immune cells and regulate immune responses. They are often regarded as the third signal for T cell activation and differentiation (13). IL-21, as an important pleiotropic cytokine, has become one of the focal points of research in immunology and organ transplantation in recent years (14–19). As a member of the type I cytokine family, IL-21 profoundly affects immune cell fate and is involved in immune regulation in diseases such as autoimmune disorders, allergies, transplant rejection, and cancer. As shown in **Figure 1**, IL-21 enhances the cytotoxic activity of NK cells and the expression of IFN-γ (20). The IL-21 signaling pathway non-redundantly promotes the differentiation of naïve T cells into Tfh cells, which assist B cells in secreting antibodies, as evidenced by a significant reduction in both the number and proportion of Tfh cells following IL-21 signaling loss (21). IL-21 particularly influences B cell fate, playing a critical and indispensable role in numerous biological processes, such as promoting germinal center responses, class switching of antibodies, differentiation of B cells into plasma cells, and ultimately leading to the formation of donor-specific antibodies (22, 23). Exogenous IL-21 significantly promotes inflammatory infiltration in the allograft, induces the proliferation of CD4^+^ T cells and B cells, and accelerates rejection in allogeneic kidney-transplanted mice (15). Moreover, some previous studies have reported elevated mRNA or protein levels of IL-21 and IL-21R in allografts, and this suggests that IL-21 is deeply involved in transplant rejection. Endomyocardial biopsies from patients with allogeneic heart transplant rejection revealed high levels of IL-21 and IL-21R mRNA (24). During acute rejection in a rat allogeneic kidney transplant model, the mRNA and protein levels of IL-21 expressed by mononuclear leukocytes were significantly increased (25). Besides, Chronic lung allograft dysfunction (CLAD) after lung transplantation is one of the major challenges in the field. A study on donor-specific antibodies (DSA) and non-HLA antibodies after lung transplantation showed that CLAD lung lymphoid aggregates with local antibodies contained larger numbers of IgG plasma cells and exhibited significantly greater IL-21 expression (26). The effects of IL-21 on immune cells will be discussed in detail in subsequent sections. In summary, IL-21 affects the effector functions of various immune cells, which play a significant role in the pathophysiological processes of transplant rejection. Therefore, IL-21 is a key mediator in transplant rejection.

This review focuses on the functions of IL-21, exploring its effects on immune cells and its role in the pathophysiological mechanisms of transplant rejection, aiming to provide potential diagnostic and therapeutic insights for clinical practice.

## 2 Structure, receptor, secretion regulation and downstream signaling of IL-21

IL-21 was discovered in 2000 (27, 28)and is classified as a type I four-α-helix bundle cytokine, sharing a common γ-chain with IL-2, IL-4, IL-7, IL-9, and IL-15. Initially, IL-21 was thought to be primarily produced by Tfh and Th17 cells (21). However, recent studies have identified peripheral helper T cells (Tph), a subset of CD4+ T cells, as another source of IL-21 production (29). The secretion of IL-21 is mediated by ROCK2 via the RhoA-ROCK signaling pathway (30). Because the IL-21 gene is adjacent to the IL-2 gene, its transcriptional levels are significantly upregulated following T cell activation, similar to IL-2. A calcium signal alone is sufficient to induce IL-21 gene expression, and this calcium signal can be blocked by calcineurin inhibitors, such as tacrolimus. NFAT or NFATc2 directly activates the transcription of IL-21, while T-bet inhibits IL-21 expression by suppressing the binding of NFATc2 to the promoter (31). Furthermore, studies have shown that the production of IL-21 is dependent on the B7/CD28 co-stimulatory pathway (32). ICOS is another critical co-stimulatory molecule that influences the binding of c-Maf to the IL-21 locus during Tfh and Th17 cell development (33, 34). c-Maf activates the promoter and enhancer regions of the IL-21 gene. Other cytokine signals also play a crucial role in regulating IL-21 expression. IL-6, IL-12, and IL-23 have been shown to effectively induce IL-21 production in CD4+ T cells (35, 36), while TGF-β, which has immune-suppressive effects, significantly inhibits c-Maf-mediated IL-21 secretion in CD4+ T cells (37, 38). These findings highlight that IL-21 expression is regulated by multiple factors and is an important cytokine in immune responses.

The specific receptor for IL-21, IL-21R, is primarily expressed in lymphoid tissues but has also been identified in other cell types, such as synovial cells and epithelial cells (39). It shares a common gamma (γ) chain with the receptors for IL-2, IL-4, IL-7, IL-9, and IL-15. Similar to other type I cytokines, such as IL-2 and IL-15, IL-21 exerts its biological functions through the JAK/STAT signaling pathway (40, 41). Upon binding to IL-21R, Janus tyrosine kinase family members JAK1 and JAK3 are activated, leading to the phosphorylation of STAT1 and STAT3, with weaker phosphorylation of STAT5. STAT proteins are present as dimeric complexes in the cytoplasm of quiescent cells and are activated by growth factors and cytokines upon receptor activation. After activation, these proteins translocate to the nucleus, where they interact with regulatory regions of target genes (40, 42, 43). The activation of STAT1 induces the transcription of a wide array of genes, including T-bet, a key regulator in Th1 lineage differentiation and immunoglobulin class switch recombination. In addition, STAT1 also plays a critical role in Tfh and Th17 responses. A study on IL-6 and STAT1 demonstrated that IL-6 signaling specifically activates STAT1 in CD4^+^ T cells, and the absence of IL-6 severely impairs Tfh differentiation (44). In another study, inhibition of STAT1-mediated transcription was shown to effectively alleviate Th1- and Th17-mediated inflammatory diseases (45). Furthermore, STAT1 is critical for the generation of T-bet+ memory B cells, which facilitate tissue-resident humoral memory by producing IgG responses upon subsequent infection (46–48). STAT3 has highly complex functions, interacting with NF-κB family members to regulate the expression of numerous cytokines and mediators such as IL-6, IL-1β, prostacyclin, and cyclooxygenase-2. These interactions play critical roles in autoimmune diseases, as well as in the induction and maintenance of pro-tumor inflammatory environments (49, 50). STAT5, on the other hand, is associated with promoting cellular transformation and preventing apoptosis (51, 52). IL-21 functions through both autocrine and paracrine mechanisms, promoting and maintaining the phenotypes of Tfh and Th17 cells (21). Additionally, the phosphoinositide 3-kinase (PI3K/Akt) pathway and the mitogen-activated protein kinase (MAPK) pathway are also critical downstream signaling pathways of IL-21. Through these pathways and their downstream genes, IL-21 participates in immune responses (43). For instance, IL-21 enhances human NK cell proliferation and effector functions, such as increased granzyme and perforin expression, via the JAK/STAT and PI3K/MAPK pathways (53). It also downregulates the expression of inhibitory receptors Ly49 and NKG2D on human NK cells by inducing STAT3 tyrosine phosphorylation (54, 55).

## 3 Effects of IL-21 on immune cells

### 3.1 Macrophage

IL-21 enhances the phagocytic capacity of primary human monocytes and GM-CSF-derived macrophages through the MAPK, PI3K-Akt, and JAK-STAT signaling pathways (56, 57), while also supporting protease activity and the expression of matrix metalloproteinase 12 (58). Macrophage polarization remains a crucial area of immunological investigation, and notably, IL-21 modulates macrophage polarization in context-dependent manners. Locally administered IL-21 in tumors has been shown to shift tumor-associated macrophages (TAMs) from the M2 phenotype towards the M1 phenotype, exerting anti-tumor effects (59). In contrast, in the presence of LPS, IL-21 decreases the expression of CD86, iNOS, and TLR4, promoting the polarization of macrophages from the M1 to the M2 phenotype (60). IL-21 also fosters M2 polarization in primary alveolar macrophages, and following lung transplantation, elevated levels of IL-21 and M2 phenotype markers have been detected in patients with idiopathic pulmonary arterial hypertension (61). In rheumatoid arthritis (RA) patients, IL-21 has been found to impair the pro-inflammatory activity of M1-like macrophages in synovial fluid and to inhibit LPS-induced secretion of inflammatory mediators by synovial fluid macrophages, thereby exerting anti-inflammatory effects (62).

During organ transplantation, the restoration of blood flow to an isolated organ triggers ischemia-reperfusion injury (IRI). A study indicated an upregulation of IL-21 expression in the graft, suggesting its potential involvement in IRI (63). It has been demonstrated that in the context of IRI, Kupffer cells shift towards the M1 phenotype, exacerbating inflammatory damage (64, 65). Besides, macrophage infiltration is closely associated with the severity and outcome of allogeneic kidney transplantation. Significant infiltration of CD68+ cells has been detected in graft biopsies from cases of acute rejection (66). Strong expression of IL-21 and IL-21R mRNA was observed in leukocytes isolated from the vascular perfusion of rat kidney allografts, with the majority of IL-21R-positive cells being monocytes. This suggests that IL-21R is induced in intravascular monocytes within the graft in response to allogeneic transplantation (25).

### 3.2 Dendritic cells

Dendritic cells (DCs), as professional antigen-presenting cells (APCs), are renowned for their powerful ability to activate naïve T cells and are considered a bridge between innate and adaptive immunity. Early in this century, studies demonstrated that IL-21 inhibits the activation and maturation of DCs, as evidenced by lower expression of MHC class II molecules, increased antigen uptake, and reduced T cell activation *in vitro* (67, 68), with similar findings observed in chickens (69). Additionally, IL-21 stimulates the secretion of granzyme B by human plasmacytoid dendritic cells (pDCs), which downregulates TLR-induced CD4+ T cell proliferation through pre-activated pDCs (70). In a type 1 diabetes model, IL-21 regulates antigen transport in DCs, facilitating the acquisition of C-C chemokine receptor 7 (CCR7), thereby promoting their migration to draining lymph nodes (71). The authors found that in a virus-induced diabetes model, DCs in the pancreatic draining lymph nodes of wild-type mice had high CCR7 expression, whereas IL-21R^-^/^-^ mice showed significantly reduced CCR7 levels. Flow cytometry revealed that CCR7^+^MHCII^+^ DCs in IL-21R^-^/^-^ mice were only about 30% of those in wild-type mice, indicating IL-21R directly regulates CCR7 expression. Although IL-21R^-^/^-^ DCs could uptake antigen normally, they failed to upregulate CCR7, reducing antigen-bearing DC migration to lymph nodes. This demonstrates that IL-21/IL-21R signaling is essential for DC migration in virus-induced type 1 diabetes, but whether this applies to transplantation requires further validation. Although similar studies are limited, this finding is important because this will cause a cascade of reactions: DCs take up antigen in presence of CCR7. Increased CCR7 expression mediates increased migration to the SLO along a CCL19/CCL21 gradient. Enhanced homing of DCs to SLO increases local T cell responses. Furthermore, IL-21 induces the apoptosis of conventional dendritic cells (cDCs) through STAT3 and Bim, a process that can be inhibited by granulocyte-macrophage colony-stimulating factor (GM-CSF) (72). ATR-107, a human anti-IL-21R monoclonal antibody, has been shown to induce DC activation and maturation, characterized by upregulation of co-stimulatory molecules such as CD86 and CD40 (73).

**Figure 2:**
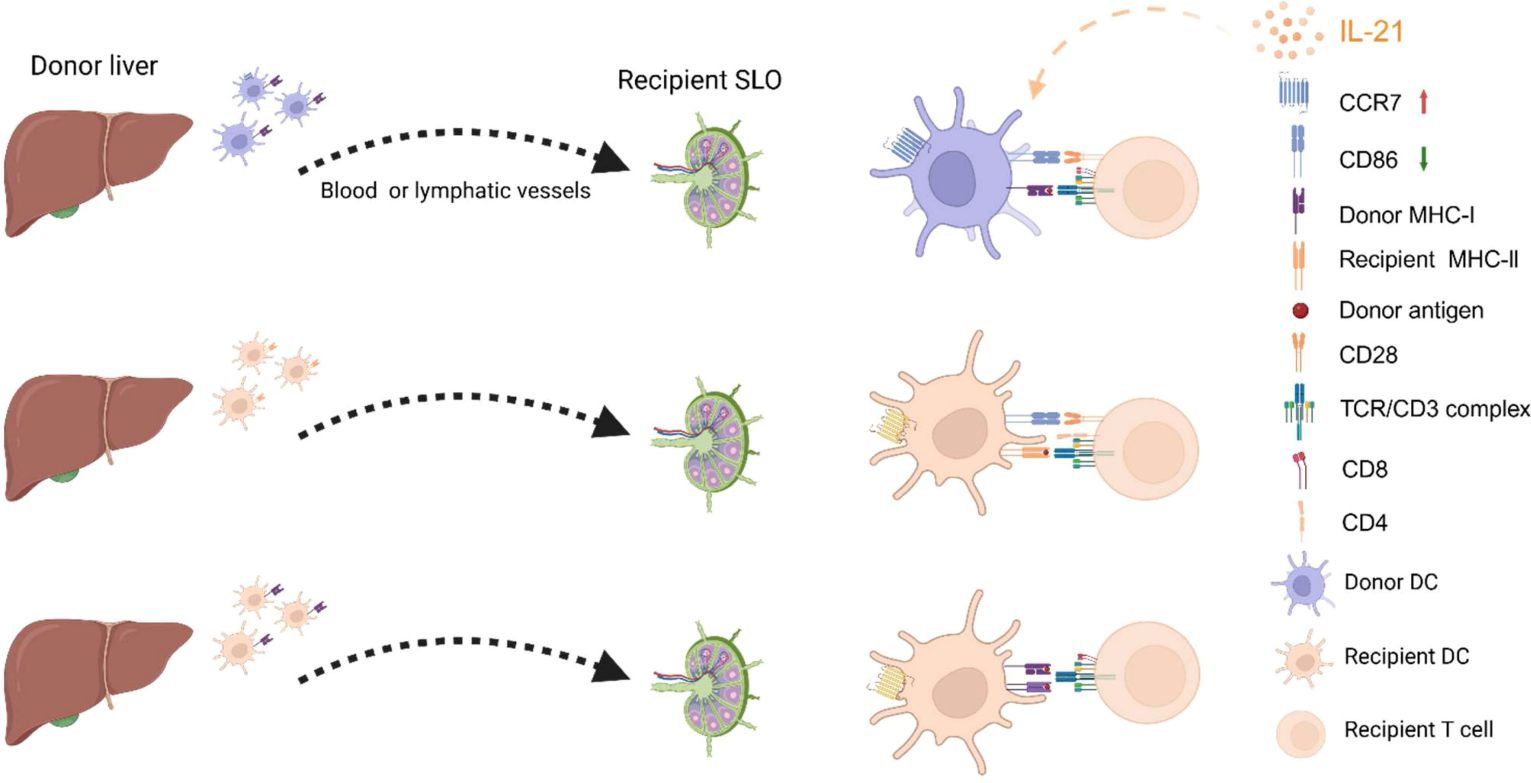
Pathways of Allogeneic Antigen Recognition and the Impact of IL-21 on DCs. From top to bottom, the figure shows the pathways of direct recognition, indirect recognition, and semi-direct recognition. On the one hand, IL-21 inhibits DC maturation by reducing the expression of surface co-stimulatory molecules such as CD86 and CD40 (not shown in the figure). On the other hand, IL-21 maintains the expression of CCR7 on DCs, enabling them to migrate to secondary lymphoid organs (SLOs). The figure was created with www.biorender.com.

The migration of DCs to secondary lymphoid organs (SLOs) following organ transplantation is essential for the presentation and recognition of allogeneic antigens. As shown in **Figure 2**, there are three well-established pathways for allogeneic antigen recognition: the direct pathway, the indirect pathway, and the semi-direct pathway (74). The direct pathway occurs when the graft vasculature connects with the recipient’s circulation, enabling donor-derived DCs to migrate directly to the recipient’s SLO, where they are recognized by recipient CD8+ T cells for their mismatched MHC class I molecules, triggering a strong acute rejection response, a phenomenon known as the “passenger leukocyte” (75). In a fully mismatched rat spontaneous allogeneic liver transplant model (PVG→DA), donor-derived DCs were found in the recipient’s celiac lymph nodes (76). The indirect pathway involves the recipient’s DCs capturing foreign antigens and migrating to the recipient’s SLO, where they present antigens via MHC class II molecules to activate CD4+ T cells that are specific for the allogeneic antigens, which is more closely associated with chronic rejection (77). The semi-direct pathway involves the transfer of donor MHC molecules to the recipient’s DCs, allowing them to present donor antigens directly to the recipient’s T cells by “cross-modifying” the surface of recipient DCs (78). Interestingly, the transfer of MHC II molecules is a bidirectional process. A previous study (79) on mouse kidney and heart transplantation found that donor and recipient MHC II molecules could be bidirectionally transferred, forming double-positive cells and facilitating crosstalk between direct and indirect antigen presentation. Another study (80) in heart transplantation showed that the semidirect pathway is crucial for CD8^+^ T cell-mediated rejection, even without donor APCs. Moreover, in hematopoietic stem cell transplantation, “cross-dressed” DCs, while unable to independently induce T cell proliferation, enhance immunological synapse formation and strengthen indirect antigen presentation (81). Thus, the migration of DCs to SLOs is a critical process, and as mentioned above, IL-21 aids DCs in acquiring CCR7. Therefore, IL-21 seems to have multifaceted effects on DCs in the context of transplant rejection.

### 3.3 NK cells

Due to the negative regulation of NK cells by MHC class I-specific inhibitory receptors, mismatched transplantation may trigger NK cell alloreactivity, resulting in graft injury and dysfunction (82). As previously mentioned, IL-21 enhances NK cell cytotoxicity and effector functions, which is generally considered a risk factor in allogeneic transplantation (20). Additionally, IL-21 promotes NK cell expansion (83), reverses NK cell exhaustion (84), and restores NK cell function in chronic HBV infection (85). Subsequent studies have shown that IL-21 has a biphasic effect on immature NK cells at different doses: low doses enhance their proliferation, while high doses reduce it (86). Interestingly, IL-21R knockout mice did not show defects in NK cell numbers or activation, and did not respond to IL-21, indicating that IL-21 is not necessary for NK cell production and development (20, 87).

In liver transplantation, NK cells display an interesting biphasic effect, with donor-derived NK cells responsible for inducing tolerance, while recipient-derived NK cells contribute to rejection (88). The latter is easy to understand, as NK cell infiltration and increased IFN-γ expression are commonly observed after transplantation (89), while NK cell exhaustion or reduced IFN-γ expression leads to improved graft survival (90). Regarding the former, a study by Marc Martínez-Llordella et al. on peripheral blood gene expression in liver transplant recipients who had discontinued immunosuppressive therapy suggested that the molecular pathways involved in the activation and effector functions of innate immune cell types (NK and γδTCR T cells) are central to maintaining operational tolerance after liver transplantation (91). In a study aimed at identifying blood biomarkers capable of predicting and diagnosing “operational tolerance” to safely reduce or even discontinue immunosuppressive therapy in liver transplant patients, the authors identified 367 differentially expressed genes in tolerant transplant recipients and further refined the gene set to 13 genes. These 13 genes exhibited the highest expression levels in patients with operational tolerance and were highly enriched in NK cells, suggesting that NK cells may play a crucial role in maintaining tolerance (92). Therefore, more research is needed to explore how IL-21 affects NK cell function and consequently influences liver transplant rejection.

### 3.4 T cells

**Figure 3:**
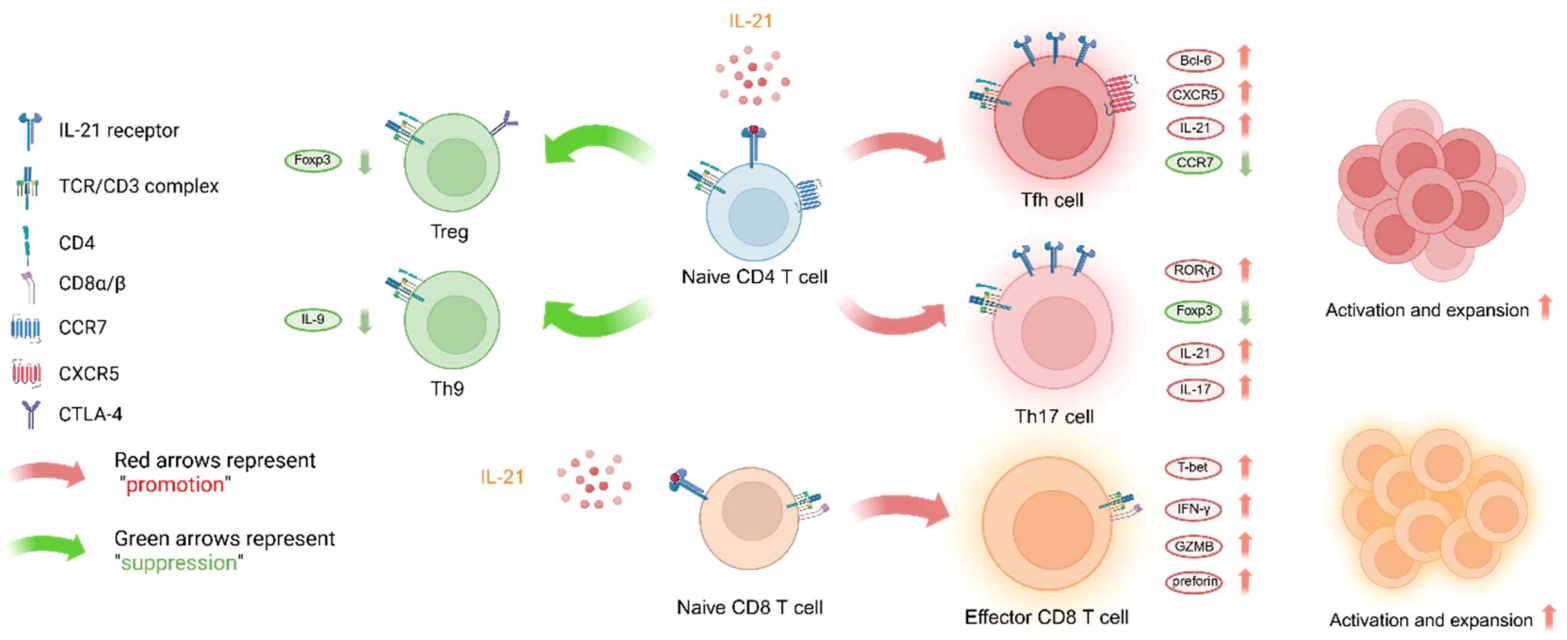
IL-21 influences T cell lineage differentiation and effector functions. Since there is limited research on the effect of IL-21 on Th22 effector function, this subset will not be discussed here. The figure was created with www.biorender.com.

T cells and B cells are the primary target cells of IL-21, and the expression of IL-21R on their cell membranes is upregulated following TCR and BCR signaling. As shown in **Figure 3**, we primarily discuss how IL-21 regulates the fate of CD4 T cell major subsets, CD8 T cells, and B cells in the context of organ transplantation.

#### 3.4.1 Tfh cells

Naive CD4+ T cells expressing chemokine receptor 7 (CCR7) are attracted to the T cell zones of lymphoid organs by the concentration gradients of CCL19 and CCL21. Upon activation by dendritic cells, these T cells upregulate CXCR5 expression and downregulate CCR7, enabling them to migrate to the T-B cell boundary of lymphoid follicles. There, they interact with B cells that recognize antigenic proteins. These activated T cells are referred to as T follicular helper (Tfh) cells (93). Tfh cells play a critical role in aiding B cells to differentiate into plasma cells, making them essential for antibody production (94). IL-21 is an extremely important cytokine for the polarization of naive T cells into Tfh cells (21). Initially, IL-21 was considered indispensable for Tfh cell development (95, 96), but subsequent studies in animals with IL-21 signaling deficiencies also revealed the formation of Tfh cells (97, 98). Nevertheless, the absence of IL-21 signaling leads to reduced levels of Bcl-6 protein and a diminished proportion of Tfh cells, as Bcl-6 is a crucial transcription factor for the differentiation and function of Tfh cells (99, 100). Bcl-6 antagonizes the transcription factors that drive the differentiation of naive CD4+ T cells into Th1, Th2, and Th17 cells (101). IL-21 produced by Tfh cells can act in an autocrine manner to maintain Tfh differentiation and in a paracrine manner to act on germinal center (GC) B cells, promoting their growth, survival, and class-switch recombination (102). Tfh cells are key players in maintaining the GC response and selecting GC B cells; without Tfh cells, the GC will collapse (103). During homologous T: B cell interactions, Tfh cells provide essential co-stimulatory signals such as CD40L-CD40 and ICOS-ICOSL to B cells, which enable B cells to undergo class-switch recombination and affinity maturation. This leads to the rapid proliferation of B cells into antibody-secreting plasma cells and memory B cells (104), generating an effective humoral immune response, but also triggering AMR in organ transplantation.

Tfh cells, which produce IL-21, are core participants in driving antibody-mediated rejection AMR by promoting B cell differentiation, proliferation, and the production of DSA (105). AMR is currently the leading cause of kidney transplant failure (106). Significant increases in circulating Tfh cells and CD86+CD38+ B cells numbers and serum IL-21 levels were observed in kidney transplant patients with AMR, and these markers were positively correlated with serum creatinine levels (107). Compared to patients without DSA, those who developed DSA after kidney transplantation had higher pre-transplant plasma IL-21 levels and increased cTfh cells (108). Another study reported that kidney transplant patients with signs of chronic rejection had a significantly higher percentage of cTfh cells and lower PD-1 expression compared to stable patients (109). Tfh cells can co-localize with B cells to promote B cell effector functions in lymph nodes and kidney grafts (19, 107, 110), resulting in chronic graft injury. In chronic AMR patients, an increase in circulating Tfh cells (cTfh, particularly cTfh2 and cTfh17) producing IL-21 is observed in blood and kidney biopsies, while follicular regulatory T cells (Tfr) are reduced (111). In the presence of IL-6 and IL-21, studies using IL-21R antagonists in co-cultures with naive T cells show a significant reduction in the proportion of Tfh cells and an increase in Tfr cells (18). In fully mismatched mouse skin graft models, lower levels of circulating DSA and reduced inflammatory cytokine levels were observed. DSA can contribute to acute rejection by forming the membrane attack complex (MAC) and can also participate in chronic rejection by inducing endothelial cell activation (112, 113). Allograft vasculopathy can indicate the occurrence of AMR. In a murine heart transplantation study, the administration of IL-21 receptor fusion protein blocked the development of chronic allograft vasculopathy in wild-type recipients of B6-bm12 heart allografts, significantly prolonging graft survival (114). Studies using IL-21R antagonists in humanized mouse skin graft models found a significant reduction in the proportion of pSTAT3-positive CD4+ T cells, fewer infiltrating CD4+, CD8+, and CD20+ cells in grafts, and significantly lower levels of skin inflammation markers such as Ker17 and Ki-67 (115). In a study of liver transplant recipients, it was found that cTfh cells helped B cells differentiate into plasmablasts in an IL-21-dependent manner *in vitro*, suggesting that cTfh cells may participate in alloreactivity after liver transplantation by aiding B cell differentiation into plasmablasts and plasma cells (116). Another study indicated that cTfh2 is associated with acute graft rejection after liver transplantation, with higher proportions of B cells associated with cTfh2 and elevated serum IL-21 levels in patients with rejection compared to those without rejection (117). In lung transplantation, AMR is also thought to be related to graft dysfunction and chronic rejection, with DSA participating in the occurrence and development of obliterative bronchiolitis syndrome (10, 118, 119).

The first-line immunosuppressant used in clinical solid organ transplantation is tacrolimus. Previous reports have shown that adding tacrolimus to *in vitro* cell cultures does not inhibit the production of Tfh cells. Specifically, after tacrolimus treatment, the number of CD4^+^CXCR5^+^ Tfh cells was reduced by only 7% compared to the control group. Tacrolimus could only partially prevent Tfh activation (CD4^+^CXCR5^+^PD-1^+^ Tfh cells were reduced by 48%) and plasmablast differentiation (120). Another study reported that pre-treatment with tacrolimus one week before transplantation significantly reduced the levels of cTfh and lymph node Tfh cells, but had no significant effect on B cells, Tfr, or Treg cells (121). Following transplantation, under tacrolimus-based immunosuppressive therapy, the cTfh levels in patients remained relatively stable (116, 122). Despite partial functional impairment, these cells still retained the ability to assist B cell differentiation into plasma cells and antibody production. These findings suggest that current immunosuppressive regimens appear to have limited efficacy in combating AMR. Considering the critical role of IL-21 signaling in Tfh cells, targeting IL-21 as a new immunosuppressive approach may represent a promising strategy.

#### 3.4.2 Th17 cells

Th17 cells are known for their high expression of IL-17, and the increased expression of IL-17 in local tissues is closely associated with allograft rejection *in vivo (* 123, 124). In kidney transplantation, exposure to IL-17 leads to the production of inflammatory mediators by renal epithelial cells (125), which induces neutrophil recruitment and contributes to acute rejection. An imbalance between Th17/Treg cells results in severe renal interstitial and tubular damage (126). In an allogeneic rat liver transplantation model, strong expression of IL-17A, IL-6, TGF-β, IL-8, and myeloperoxidase (MPO) was observed in the grafts, and the proportion of Th17 cells in circulation significantly increased, indicating that Th17 cells contribute to liver allograft rejection in rats (127). Peripheral blood analysis of patients six months after liver transplantation showed that the Th17/Treg ratio in stable recipients was similar to pre-transplant values, while it was significantly elevated in patients with acute allograft rejection, suggesting that the Th17/Treg ratio may be a predictive factor for acute rejection (128).

Th17 cells are also a major source of IL-21, with their IL-21 mRNA and protein levels being five times higher than those of Th1 and Th2 cells (129). A study on kidney allografts from patients experiencing rejection found that Th17 cells accelerate graft destruction by promoting lymphocyte neogenesis through IL-21 production. Complement activation and high IL-21 expression were detected in grafts with high levels of Th17 infiltration (130). IL-21 plays a crucial role in Th17 effector functions, promoting CD4^+^ T cell proliferation and maintaining Th17 cell identity by upregulating RORγt and downregulating Foxp3, thereby inhibiting Treg differentiation (131–133). Th17 cells also assist B cell activation, facilitating the generation of antigen-specific B cells and promoting antibody class switching to IgG1, IgG2a, IgG2b, and IgG3 (134). In IL-21R knockout mice, Th17 frequencies decrease and the response is defective (129, 135).

The role of IL-21 in Th17 differentiation remains controversial. While both IL-21 and TGF-β are considered essential for human Th17 cell differentiation (136), studies in mice suggest otherwise. In murine models, IL-6, IL-1β, and TGF-β are the primary cytokines driving naïve CD4^+^ T cells toward the Th17 lineage, with IL-21 being dispensable. In IL-21 and IL-21R knockout mice, IL-6-induced Th17 differentiation occurs independently of IL-21 and is even stronger than IL-21-induced differentiation *in vitro (* 137), These findings indicate that while IL-21 is not required for Th17 differentiation in mice, it remains a critical factor for Th17 cell function.

#### 3.4.3 Treg cells

As mentioned earlier, IL-21 can downregulate the expression of Foxp3 in CD4 T cells, and Foxp3 is one of the important transcription factors and markers for Treg differentiation. Therefore, IL-21 can directly impair Treg homeostasis. In addition, IL-21 inhibits the production of IL-2 in T cells. Although conventional T cell responses are not impaired because IL-21 can substitute for IL-2 as a T cell growth factor, IL-21 cannot replace IL-2 in supporting the Treg compartment. Thus, IL-21 signaling in conventional T cells can indirectly affect Treg homeostasis by reducing the availability of IL-2 (138).

In organ transplantation, Treg cells are generally considered beneficial for promoting transplant tolerance. Treg cells secrete CD25, which depletes IL-2 availability in other lymphocytes, thereby inhibiting the survival of surrounding T cells (139). Additionally, they can suppress effector T cell activation by competing with CD28 for binding to CD80/CD86 through their surface immune checkpoint molecule CTLA-4. Alessandra et al. reported that IL-21 signaling enhanced T cell signaling and mixed lymphocyte responses in T cells treated with IL-21 pOrf plasmid and stimulated with anti-CD3/anti-CD28, suppressed Treg generation and function, and observed strong immune tolerance in a mouse islet transplant model after IL-21R blocking agent and CTLA-4 globulin treatment (140). The study by Yeqi Nian and colleagues (18) showed that treatment with an IL-21R antagonist in mice after allogeneic skin transplantation significantly reduced the Tfh/Tfr ratio in their splenocytes, thereby lowering the levels of donor-specific IgG antibodies and significantly inhibiting AMR. Christoph Bucher et al. (141)reported that IL-21 inhibited the differentiation of inducible Tregs and further suggested that IL-21 blockade is a promising target to reduce graft-versus-host disease (GVHD) damage. The maturation of dendritic cells in the liver is much lower than in peripheral lymphoid organs, manifested by low expression of MHC-II, CD80/CD86, and high expression of TGF-β, which favors the expansion and functional maintenance of Tregs (142–144). In patients without rejection, the Treg population gradually returns to normal levels over time, whereas in patients with acute rejection, it remains at lower levels (145), suggesting that Tregs are associated with the fate of allogeneic liver grafts. Therefore, from the perspective of Tregs, IL-21 is an anti-tolerance cytokine antagonizes Treg-induced immune tolerance.

#### 3.4.4 Th1 and Th2 cells

Since Th1 and Th2 cells are the earliest classified CD4 T cell subpopulations and antagonize each other in driving naive CD4 T cell differentiation toward the opposing lineage (146), this section discusses both subpopulations together. Early studies reported that IL-21 can upregulate the transcription of Th1-related genes and promote Th1 responses (147), which is understandable because IL-21 is a strong activator of STAT1, which in turn upregulates the Th1-related transcription factor T-bet. However, later studies indicated that IL-21 is a Th2 cytokine, which specifically inhibits the production of IFN-γ by Th1 cells and amplifies the Th2 response (148). Additionally, contradictory reports regarding IL-21’s role in Th2 responses have emerged. One study stated that in an OVA-induced airway inflammation model, IL-21 receptor signaling is an indispensable part of the development of Th2 effector responses, mediating Th2 cell survival or migration to peripheral tissues. However, IL-21R signaling is not required to control the development of severe Leishmania infection, suggesting it may be redundant in Th1 responses (149). Another study in the same OVA-induced airway inflammation model showed that IL-21 reduced cytokine production polarizing toward Th2 and induced Th2 cell apoptosis by downregulating Bcl-2 (150). It is well known that Th2 cells primarily contribute to the development of parasitic infections and allergic diseases, in which IL-21 plays a dual role. IL-21 can promote allergic airway inflammation by driving apoptosis of FoxP3(+) regulatory T cells (151), but it can also suppress IL-4-mediated IgE secretion (152), further exemplifying IL-21’s multifunctionality.

It is widely acknowledged that Th1 responses dominate in solid organ transplants. Various inflammatory cytokines produced by Th1 cells, such as TNF-α and IFN-γ, are hallmark markers of transplant rejection and can mediate target tissue damage (153). Considering that IL-21 primarily participates in regulating the fate of Tfh and Th17/Treg cells, but not as a definitive cytokine for Th1/Th2 lineage differentiation, this section does not further discuss it.

#### 3.4.5 Th9 cells

Th9 cells are defined by the secretion of IL-9 (154) and primarily exert effects in parasitic infections, allergic reactions, and certain autoimmune diseases. It has been shown that IL-2-JAK3-STAT5 signaling is essential for Th9 differentiation, while IL-21 inhibits Th9 differentiation. This difference is mediated by Bcl-6, whose increased expression suppresses IL-9 production (155). An early study (156) had shown that IL-21 could support the polarization of human Th9 cells in the presence of IL-4 and TGF-β, which indicated the effect of IL-21 on Th9 differentiation may exhibit species-specific differences.

To some extent, Th9 cells are believed to induce immune tolerance, which is beneficial in transplantation. IL-9 secreted by Th9 cells can recruit mast cells and promote their proliferation. Mast cells can negatively regulate immune responses through surface co-inhibitory molecules and secretion of immunosuppressive factors, such as IL-4 and IL-10, to maintain immune homeostasis (157). Additionally, mast cells are essential in Treg-dependent peripheral immune tolerance, and neutralization of IL-9 significantly accelerates allograft rejection in mice (158). Current research findings do not support the involvement of Th9 and IL-9 in kidney transplant rejection. No detectable IL-9 gene expression has been observed in allogeneic kidney transplant tissues. Additionally, a study on kidney transplantation in rats reported that IL-9 is expressed in normal kidneys but is nearly undetectable during rejection, while IL-9Rα is barely expressed in normal kidney tissue (159). A similar conclusion was drawn in a mouse heart transplantation model. L.F. Poulin et al. observed that allogeneic heart grafts were rejected within nine days in both wild-type and IL-9 knockout mice (160). In a study by E. Fábrega and colleagues on 30 liver transplant recipients who had not experienced rejection for 8 years, their serum IL-9 levels were significantly higher than those of healthy controls (n=30) (161). They then conducted another study dividing 50 liver transplant recipients into two groups: Group I, consisting of 15 patients with acute rejection, and Group II, consisting of 35 patients without acute rejection. On postoperative days 1 and 7, there were no significant differences in serum IL-9 levels between Group I and Group II. However, the serum IL-9 levels of all 50 liver transplant recipients remained higher than those of healthy controls (n=34), suggesting that IL-9 may be only minimally involved in acute liver transplant rejection (162).

#### 3.4.6 CD8 T cells

It is well-known that CD8 T cells play a crucial role in anti-tumor immunity, and IL-21 can effectively enhance this effect (163). However, CD8 T cell-mediated rejection (TCMR) is a key feature of acute rejection after allogeneic transplantation. Naïve CD8 T cells recognize alloantigens presented on MHC class I molecules by APCs (antigen-presenting cells) in secondary lymphoid organs. Upon co-stimulation with APCs, they become activated, exit the secondary lymphoid organs, enter the circulation, and migrate to local tissues to eliminate antigen-expressing target cells, resulting in graft injury. The degree of CD8 T cell infiltration into the graft is often considered a measure of the severity of acute rejection (164–166). Previous studies have reported that using spleen cells from IL-21R knockout mice significantly reduced the incidence and mortality of GVHD (graft-versus-host disease) after bone marrow transplantation (167). In addition, in a study on xenogeneic graft-versus-host disease (X-GVHD) (168), the authors found that in mice treated with IL-21 monoclonal antibody after receiving human PBMCs, the number of CD8 T cells in peripheral blood and spleen decreased, while the number and proportion of Treg cells in the spleen increased. This suggests that IL-21 blockade may be an attractive strategy in X-GVHD. Vinh Nguyen et al. (169) later used IL-21R-deficient donor T cells to induce acute GVHD and found a reduction in peak donor CD8 T cell numbers and a decrease in CTL effector functions. This further suggested that effector CTL maturation depends on IL-21R signaling in antigen-specific donor CD8 T cells. Memory T cells, due to their lower activation threshold, can respond rapidly upon reencounter with the antigen, mediating acute rejection. Overexpression of IL-21 has been shown to promote the accumulation of a large number of CD8^+ memory T cells, reducing the proportion of naïve T cells (170, 171).

IL-21 promotes CD8 CTL (cytotoxic T lymphocyte) activity through the transcription factor T-bet (172), making it an important signal for CD8 T cell expansion and effector functions (173, 174). The expression of IL-21R on the surface of CD8 T cells enhances their activation (175). In an allogeneic mixed lymphocyte reaction, IL-21 was observed to effectively expand both memory (CD44^high) and naïve (CD44^low) CD8 T cells, increasing IFN-γ production *in vitro*. However, in IL-21R knockout mice, CD8 T cell expansion and cytotoxicity were impaired (176). During chronic viral infections, IL-21 derived from Tfh cells maintains the effector CD8 T cell response (177, 178).

### 3.5 B cells

**Figure 4:**
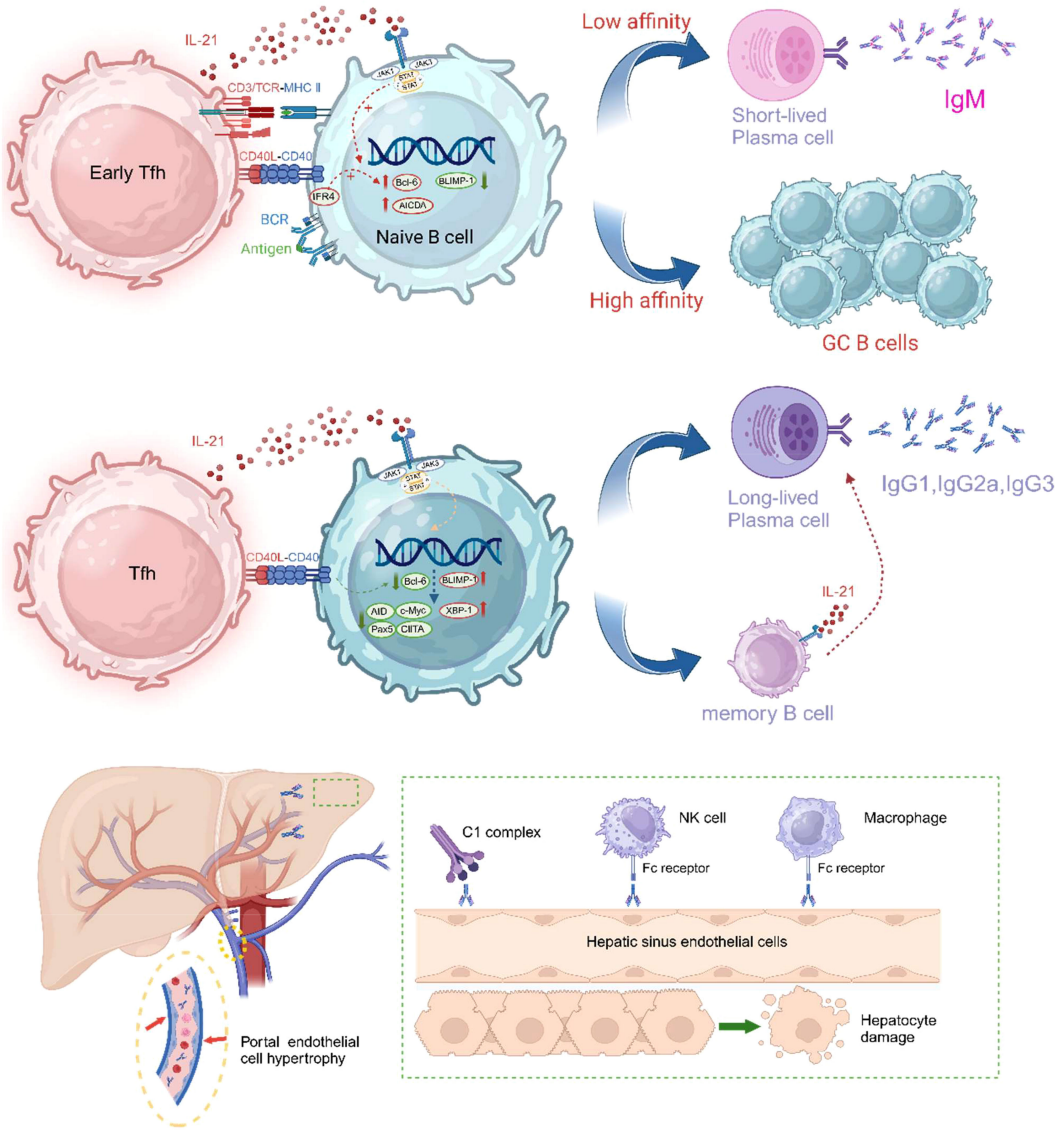
IL-21 promotes the formation and maintenance of germinal centers and plays a decisive role in the differentiation of GC-derived B cells into plasma cells. The CD40L-CD40 signaling pathway has been shown to work synergistically with IL-21 to promote Blimp-1 activation and plasma cell differentiation. CD40 signaling enhances STAT3-driven Blimp-1 upregulation by reducing Bcl-6 expression. Other co-stimulatory signals, such as ICOS-ICOSL and CD28-CD80/86, also promote plasma cell differentiation. Subsequently, DSA can injure endothelial cells by activating the complement system or through ADCC (antibody-dependent cellular cytotoxicity). In liver transplant patients experiencing AMR, hypertrophy of portal vein endothelial cells, obstructive portal vein disease, and microvasculitis are commonly observed. The drawing was inspired in part by Aldo J. Montano-Loza et al. (183). The figure was created with www.biorender.com.

IL-21 has a significant impact on the fate of germinal centers (GCs) and B cells. Germinal centers (GCs) are specialized anatomical regions within lymphoid organs where activated B cells undergo high-frequency somatic mutations, antibody affinity maturation, rapid proliferation, and produce memory B cells and long-lived antibody-secreting plasma cells. IL-21 signaling promotes the formation and maintenance of GCs. IL-21 enhances the synergistic induction of c-MYC and phosphorylated ribosomal protein S6 by B cell receptor (BCR) and CD40 (179–181). Both c-MYC and S6 are part of the mTOR pathway, a core pathway for cell growth and metabolism that is essential for GC B cells to respond effectively and support their rapid and extensive clonal expansion (182). As shown in **Figure 4**, IL-21 signaling significantly increases and maintains the expression of the key transcriptional regulator of GC B cells, Bcl-6 (97, 98, 184). Bcl-6 is indispensable for GC formation, and GC B cells lacking Bcl-6 cannot migrate to the lymphoid follicle center to differentiate into long-lived plasma cells (185). Although GCs can form without IL-21 signaling, mice deficient in IL-21 or IL-21R expression have defective GC structures, reduced numbers of GC B cells (98), and decreased B cell proliferation (18, 186), while plasma cell differentiation requires extensive B cell clonal expansion (187). GC B cells undergo AID-mediated somatic hypermutation, and only those B cells with high affinity are positively selected, then differentiate into plasma cells with the help of Tfh cells. IL-21 has been identified as a key differentiation factor for plasma cells derived from GCs and promotes the expression of the plasma cell master transcription factor B lymphocyte-induced maturation protein 1 (BLIMP1) (97, 186, 188). BLIMP1 further suppresses the expression of genes such as AID, Bcl-6, PAX5, and c-Myc, ultimately terminating the GC program and positively regulating XBP1 expression to prepare B cells for antibody production and secretion (189). IL-21 induction of Bcl-6 and Blimp-1 expression appears to be contradictory, in fact, IL-21 exerts a spatiotemporal effect on B cells, making its regulation of these factors dynamic. Initially, IL-21 activates STAT3, enhancing the expression of Bcl-6, which supports the proliferation of GC B cells. At this stage, Bcl-6 dominates, allowing GC B cells to continue proliferating and undergo somatic hypermutation in the germinal center. As the immune response progresses, IL-21 drives B cell differentiation into plasma cells through STAT3-mediated upregulation of Blimp-1. In the later stages of the immune response, IL-21 induces Blimp-1, promoting the differentiation of high-affinity B cells into long-lived plasma cells or memory B cells, ensuring long-term immune protection. Additionally, IL-21 signaling upregulates the expression of CD86 on the surface of B cells (190), further enhancing T:B co-stimulatory signals. Interestingly, in the absence of BCR signaling and T cell co-stimulation, IL-21 stimulation can induce B cell apoptosis, which is thought to be related to the elimination of self-reactive B cells (191). Moreover, Breg cells, a subset of B cells with immunoregulatory functions, have recently been found to develop and expand in response to IL-21 and CD40 signaling, specifically driving the development of the B10 subset (IL-10 secreting Breg cells) (192), a function that has been subsequently confirmed in research (193, 194).

In solid organ transplantation, alloreactive B cells are the direct source of DSA. Combined with the previously discussed role of Tfh cells in AMR, the importance of IL-21 in the pathophysiology of AMR is self-evident. Kitty de Leur et al. reported that IL-21R blockade inhibited plasma cell differentiation, significantly reducing levels of IgM and IgG2 (195). Nian, Y et al. verified in a fully mismatched mouse skin transplant model that IL-21R blockade induced defects in germinal center structures and reduced DSA production (18). Van Besouw NM et al. suggested that the number of IL-21 producing donor-specific cells before and after transplantation could predict renal transplant rejection (196). In a study of serum cytokines in liver transplant patients, it was found that in the group with acute rejection, IL-21 mRNA expression patterns and serum IL-21 levels showed a steady increase, suggesting that IL-21 exhibits pro-inflammatory characteristics in liver transplant rejection patients (197).

Tacrolimus has limited effects on B cell proliferation and antibody production, whereas mycophenolic acid and rapamycin are more potent in this regard (198). Therefore, targeting the IL-21 signaling pathway may compensate for tacrolimus’ insufficiency in suppressing AMR. Some research teams have explored strategies to enhance tacrolimus-based immunosuppression by targeting downstream signaling pathways involved in T cell and B cell activation. Park JS et al. (199)reported that combining a STAT3 inhibitor with tacrolimus increased Treg numbers in lupus-susceptible mouse spleen cells while decreasing the proportions of GC B cells and plasma cells. Tobias Deuse et al. (200) demonstrated that the JAK1/JAK3 inhibitor R507, when combined with tacrolimus, had a synergistic effect, significantly prolonging the survival of allogeneic heart transplants compared to tacrolimus alone and reducing donor-specific IgM levels. While these studies do not specifically target IL-21 signaling, they highlight the potential of modulating JAK-STAT pathways to improve immunosuppressive efficacy. Given that IL-21 signals predominantly through JAK1 and STAT3, direct blockade of the IL-21 pathway may represent an additional avenue for combination therapy to enhance tacrolimus’ efficacy in preventing AMR.

## 4 Clinical trials related to IL-21

Clinical studies involving the IL-21 signaling pathway as an intervention in the field of organ transplantation are relatively limited, but significant work has been done in the fields of autoimmune diseases and cancer immunotherapy. The safety and tolerability of NNC0114-0006 in SLE patients were evaluated in a Phase I trial (NCT01689025), with researchers aiming to assess the incidence of adverse events (AEs) and pharmacokinetic data (201). Unfortunately, this clinical study was forced to terminate due to the enrollment of only 10 participants. ATR-107 is a fully humanized monoclonal antibody targeting IL-21R (202). Its pharmacodynamic studies have shown positive results. Intravenous administration of 60mg or 120mg can maximize the occupancy of IL-21 receptors, and this occupancy effect can persist for up to 42 days after a single dose. Unfortunately, 76% (35 out of 46) of the subjects receiving ATR-107 developed anti-ATR-107 antibodies (ADA). These ADAs were detected between 42- and 105-days post-administration. The high immunogenicity of ATR-107 is considered a major obstacle in its drug development. NNC01140006 is a monoclonal antibody targeting IL-21. Previous safety trials indicated that it was well tolerated in healthy subjects and RA patients, with initial signs of reduced RA activity observed in the 25 mg/kg dose group (203). Subsequently, a multicenter, randomized, parallel-group, placebo-controlled, double-blind, phase 2 clinical trial aimed at evaluating the protective effect of NNC01140006 combined with liraglutide on β-cell function in adults with recent-onset type 1 diabetes showed that at week 54, the MMTT-stimulated C-peptide concentration in the combination treatment group decreased by 10% (ratio to baseline 0.90), significantly better than the 39% decrease in the placebo group (ratio 0.61). The decrease in all active treatment groups at week 54 (approximately 0.50 percentage points) was greater than in the placebo group (0.10 percentage points), but the difference was not statistically significant. No adverse events related to the trial treatment were observed throughout the trial. This suggests that the combination of anti-IL-21 antibody and liraglutide may help protect β-cell function in adults with recent-onset type 1 diabetes, with good safety, and warrant further evaluation (204). BOS161721 is a humanized IgG1 monoclonal antibody with three mutations (M252Y/S254T/T256E) that inhibits IL-21 bioactivity. It has been shown to be well-tolerated at various doses, dose-dependently inhibiting IL-21-induced pSTAT3, and reversing the downregulation of genes crucial for IL-21-induced tolerance and T-cell exhaustion (205). The subsequent clinical trial (206) investigating the efficacy of BOS161721 in SLE patients has been completed. However, based on the data currently provided by the researchers, the trial results do not appear to be particularly promising. There was no significant difference in the SRI-4 response, which is associated with improved outcomes, between the placebo group and the BOS161721 120 mg treatment group (hereinafter referred to as the treatment group) (51.4% vs. 53.3%, p=0.8434). However, the difference between the placebo group and the treatment group was significant in terms of the absence of new severe disease activity (73.0% vs. 90.7%, p=0.0141). Therefore, further trials may be necessary to validate the efficacy of BOS161721. In patients with metastatic melanoma and renal cell carcinoma, intravenous infusion of recombinant human IL-21 resulted in upregulation of perforin and granzyme B mRNA levels in CD8 T cells and NK cells (207). A Phase II study in metastatic melanoma patients confirmed the biological activity of IL-21, with 9 of 40 patients showing partial remission and 16 showing stable disease (208). Another study on metastatic melanoma patients showed that IL-21-induced polyclonal CTL, in combination with CTLA4 blockade, controlled refractory metastatic melanoma. This combination therapy achieved durable complete remission in patients where both monoclonal CTL and anti-CTLA4 therapy had previously failed (209). When recombinant human IL-21 was used in combination with the monoclonal antibody cetuximab, which targets the epidermal growth factor receptor, enhanced anti-tumor activity was observed, with serum sCD25 levels increasing in a dose-dependent manner, and 60% of patients achieving stable disease (210). While recombinant IL-21 has been investigated in cancer patients to enhance anti-tumor immunity, in transplantation, IL-21 is more likely to contribute to allograft rejection. Therefore, in contrast to oncology settings where IL-21 administration is beneficial, transplantation research is supposed to focus on IL-21 blockade as a potential immunosuppressive strategy. These tumor-related clinical studies have demonstrated that IL-21 can activate CD8+ T cells and NK cells (upregulating perforin and granzyme B) and induce polyclonal CTL responses, a mechanism similar to IL-21-driven alloreactive T cell activation in transplant rejection. Therefore, researchers focusing on transplant immunology may find inspiration from the interventions used in these clinical studies. For instance, this work (209) showed promising results with exogenous IL-21 combined with CTLA-4 blockade therapy. This naturally raises the question of whether combining IL-21 blockade with a CTLA-4-related drug already used in transplantation, such as Belatacept (Previous work (120) had shown that Belatacept has a limited effect in the generation of both Tfh cells and donor antigen-driven plasmablast), could yield more favorable results in suppressing transplant rejection. Although this idea has been validated by researchers in a murine allogeneic islet transplantation model with positive results (140), more data are still needed for support. We believe it could serve as similar inspiration for readers.

## 5 Conclusion

IL-21 plays a crucial role in transplant rejection as a key immunomodulatory factor. This review comprehensively discusses the IL-21 signaling pathway, its role in immune cell function regulation, and how these immune cells contribute to the pathophysiology of transplant rejection. IL-21 is not only a critical factor for the development and functional maintenance of Tfh and Th17 cells but also plays a central role in the regulation of immune effector cells such as B cells and NK cells. Its role in transplant immune responses is complex and multifaceted.

The biological function of IL-21 is mediated by its receptor, IL-21R, and it affects target cells through the JAK/STAT signaling pathway. IL-21 activates STAT1, STAT3, and other signaling pathways to induce the activation and expansion of CD8 T cells, enhancing their cytotoxicity. Similar effects are observed in NK cells, enhancing their immune surveillance of transplant tissues, thereby participating in acute transplant rejection. Additionally, IL-21 is essential for the differentiation and maintenance of CD4 T cells, particularly Tfh and Th17 cells, which play significant roles in AMR. IL-21 influences B cells by promoting the clonal expansion of germinal center B cells and is indispensable for the terminal differentiation of B cells into plasma cells. Therefore, IL-21 not only exacerbates T cell-mediated rejection (TCMR) through its effects on cellular immune responses but also drives AMR by enhancing humoral immune responses.

The role of IL-21 in transplant immunity suggests that it may become a potential therapeutic target. Interventions targeting the IL-21 signaling pathway, such as using IL-21R antagonists or IL-21 antibodies, may provide new strategies for treating transplant rejection. Such interventions could inhibit excessive immune responses, reduce the incidence of rejection, and promote transplant organ tolerance. Furthermore, existing immunosuppressive protocols, such as those based on tacrolimus, seem limited in their effect on AMR. Therefore, combining tacrolimus with IL-21 signaling blockade may be a promising therapeutic approach.

In summary, IL-21 plays a multifaceted role in transplant rejection. The complexity of the IL-21 signaling pathway offers an opportunity for in-depth research on immune regulation. Further studies will help elucidate its specific role in transplant immunity and drive the development of IL-21-targeted immunotherapy strategies. In the future, therapies targeting IL-21 signaling may offer new directions for improving long-term transplant organ tolerance and enhancing the clinical outcomes of transplantation.
